# A short form of the Spanish version of the Netherlands empowerment list: development and validation

**DOI:** 10.1007/s00127-025-02970-9

**Published:** 2025-07-30

**Authors:** Hernán María Sampietro, Georgina Guilera, Ángela I. Berrío, Maite Barrios, J. Emilio Rojo, Juana Gómez-Benito

**Affiliations:** 1 ActivaMent Catalunya Associació, Barcelona, Spain; 2 Universitat de Barcelona, Barcelona, Spain; 3 Universitat Internacional de Catalunya, Barcelona, Spain

**Keywords:** Mental health, Empowerment, Short-form, Psychometric properties

## Abstract

**Purpose:**

The Netherlands Empowerment List (NEL) is the only psychometric instrument for evaluating empowerment that has been adapted and validated for use in the Spanish-speaking population. Although the Spanish NEL has demonstrated good psychometric properties, it is relatively lengthy. This study aimed to develop and validate a short form of the Spanish NEL.

**Method:**

The sample comprised 406 users of community mental health services, split into two groups. With the development subsample (*n* = 200), we developed a short form of the Spanish NEL based on the factor loadings obtained through exploratory factor analysis. The validation subsample (*n* = 206) was then used to evaluate its psychometric properties (internal structure, differential item and test functioning by gender, internal consistency, temporal stability, and validity based on relationships with other variables). Additionally, percentile ranks for each raw score were calculated using the total sample (*n* = 406).

**Results:**

We developed a 12-item short form of the Spanish NEL, confirmed its unidimensionality, and demonstrated that the scale scores had excellent internal consistency and temporal stability. Scores on the Spanish NEL-12 also correlated strongly with measures of empowerment, recovery, hope, and perceived social support.

**Conclusion:**

The results suggest that scores on the Spanish NEL-12 provide a valid and reliable measure of empowerment in Spanish-speaking users of mental health services. Furthermore, the study provides preliminary percentile scores to support its use in clinical settings for evaluating empowerment.

**Supplementary Information:**

The online version contains supplementary material available at 10.1007/s00127-025-02970-9.

## Introduction

The World Health Organization (WHO) defines empowerment as a “multidimensional social process through which individuals and groups gain a better understanding and control over their lives” [1]. This concept was introduced into the field of mental health by the movements of users and survivors of psychiatry. Promoting empowerment was one of the foundational objectives of these movements, alongside offering mutual support and defending the rights of people who have been psychiatrized [2]. From an academic standpoint, and particularly in the development of community mental health care, empowerment was rapidly adopted as a primary goal of professional care [3]. It has also been central to recovery-oriented practice in mental health, which from the outset has emphasized that control and choice are fundamental to an individual’s recovery journey [4]. Accordingly, empowerment is now considered a crucial component of the conceptual model used in research and practice on recovery, known as the CHIME framework, the acronym being derived from the five dimensions or key elements of the recovery process: Connectedness, Hope, Identity, Meaning, and Empowerment [5].

The growing acceptance and recognition of the importance of empowerment led to it becoming a cross-cutting objective in the mental health strategic plans of the WHO [6, 7]. In fact, both the WHO and the United Nations now promote recovery-oriented and rights-based mental health systems which prioritize people’s empowerment and the active participation of individuals in their own recovery process [8]. This reflects the United Nations Convention on the Rights of Persons with Disabilities, which stipulates that States Parties should prioritize actions to protect and promote their legal capacity, community inclusion, autonomy, citizenship, and empowerment [9]. These international directives have led to the progressive integration of empowerment as a strategic objective in public mental health policies around the world, and it is increasingly recognized as a core value in high-quality, patient-centered care [10]. Consequently, empowerment is now considered not only as a means to promote recovery but also an essential goal in itself, and hence it is a care outcome that needs to be measured [10].

To address this need, several instruments aimed at evaluating the empowerment of mental health service users have emerged in recent decades. One such instrument is the Netherlands Empowerment List (NEL) [11], which offers numerous advantages over earlier scales [12–16] that were often criticized for significant limitations. For instance, two systematic reviews — one by Barr et al. [17] and another by Pekonen et al. [10]— highlighted the low methodological quality of many studies and the lack of comprehensive psychometric testing of the instruments being described. By contrast, the NEL was developed in accordance with the COSMIN criteria [18], and it has demonstrated good psychometric properties [11]. A recent study in which the NEL was translated and adapted for use in the Spanish-speaking population likewise reported robust psychometric properties, similar to those of the original version [19].

Although NEL scores have been shown to be both valid and reliable, the fact that it is a 40-item scale can make it cumbersome to administer. In practice, shorter scales are often necessary to reduce respondent burden in a battery of instruments, to improve usability [20], and to facilitate research that might otherwise be infeasible due to time constraints [21]. However, reducing the number of items should not be done at the expense of a significant loss in measurement precision [22]. With this in mind, the aims of the present study were to create a shortened version of the Spanish NEL and to provide evidence of validity and reliability for its scores.

## Method

### Participants

The sample for this study was the same as that used in validating the Spanish NEL [19]. All participants were adults (≥ 18 years old) and users of community mental health services (*n* = 406); that is, individuals diagnosed with severe mental disorders who receive daily or weekly professional support through individual and group activities to promote personal and functional recovery, but who are not experiencing an acute or subacute mental health crisis. The exclusion criteria were the presence of relevant cognitive impairments, comprehension difficulties, or severe and decompensated somatic disease.

### Instruments

*Spanish version of the Netherlands Empowerment List* (Spanish NEL) [19]. This scale is an adaptation of the NEL [11] and comprises 40 items rated on a 5-point Likert-type scale (1 = Completely disagree; 5 = Completely agree). The original NEL had a six-factor structure encompassing the following domains: confidence and purpose, self-management, connectedness, social support, caring community, and professional help. It demonstrated excellent internal consistency (Cronbach’s α = 0.94), good test-retest reliability (ICC = 0.79), correlation with other variables in the expected direction and magnitude, and good responsiveness [11]. The Spanish NEL displayed an excellent fit to both the original six-factor structure and a one-factor second-order model comprising six first-order factors and one second-order general factor. Its total score showed excellent internal consistency (McDonald’s ω = 0.98, Cronbach’s α = 0.96), high temporal stability (ICC = 0.86), and strong positive correlations with scores on the Empowerment Scale [14], the Maryland Assessment of Recovery Scale [23], the Dispositional Hope Scale [24], and the Multidimensional Scale of Perceived Social Support [25, 26].

*Maryland Assessment of Recovery Scale* (MARS-12) [23]. This is a 12-item scale designed to measure recovery across six factors: self-direction/empowerment, holistic, non-linear, strengths-based, responsibility, and hope. Each item is rated using a 5-point Likert-type scale (1 = Not at all; 5 = Very much). The recently validated Spanish version demonstrated adequate psychometric properties [27]. In our sample, the MARS-12 total score exhibited excellent internal consistency (McDonald’s ω = 0.95; Cronbach’s α = 0.95).

*Dispositional Hope Scale* (DHS) [24]. This scale comprises 12 items (four of which are fillers) distributed across two factors: pathway and agency (four items each). Items are rated using a 4-point Likert-type scale (1 = Definitely false; 4 = Definitely true). The validated Spanish version showed appropriate psychometric properties [28], and in the present sample, the DHS total score displayed excellent internal consistency (McDonald’s ω = 0.92; Cronbach’s α = 0.93).

*Multidimensional Scale of Perceived Social Support* (MSPSS) [25, 26]. This 12-item scale assesses perceived social support across three factors: family, friends, and significant others. The MSPSS employs a 7-point Likert-type scale (1 = Very strongly disagree; 7 = Very strongly agree), and good reliability and validity were reported for the Spanish adaptation [29]. In our sample, the MSPSS total score likewise showed excellent internal consistency (McDonald’s ω = 0.98; Cronbach’s α = 0.94).

For all the above scales, total scores are calculated by summing the scores of all items (with the exception of the DHS filler items), and a higher score indicates a higher level of the variable being assessed.

### Procedure

All users of 17 community mental health services who met the inclusion criteria were invited to participate, and all those who provided informed consent were included in the study.

This study was conducted in two phases. Phase 1 was aimed at developing a shortened version of the Spanish NEL based on the factor loadings of its items, while Phase 2 involved evaluating the psychometric properties of this short form.

To this end, we split the sample into a development subsample and a validation subsample. The former consisted of 200 participants with a mean age of 47.29 years (*SD* = 10.02; range 20–68), while the latter comprised 206 participants with a mean age of 48.34 years (*SD* = 9.65; range 20–71).

All participants were invited to attend a retest session one or two weeks after the first data collection to assess temporal stability. Of these, 66 attended the second session, completed the Spanish NEL again, and were directly assigned to the validation subsample. All other participants for both subsamples were randomly selected until the pre-established sample size was reached. The sociodemographic characteristics of both subsamples are presented in Table 1.

**Table 1:** Sociodemographic characteristics of the development and validation subsamples and the total sample

	Development subsample (*n* = 200)	Validation subsample (*n* = 206)	Total sample (*n* = 406)
Age, *M (SD)*	47.3 (10.0)	48.3 (9.7)	47.8 (9.8)
Age range	20–68	20–71	20–71
Gender *n* (%)			
Male	101 (50.5)	112 (54.4)	213 (52.5)
Female	98 (49.0)	93 (45.1)	191 (47.0)
Non-binary	1 (0.5)	–	1 (0.3)
Not answered	–	1 (0.5)	1 (0.3)
Diagnosis^1^ *n* (%)			
Anxiety disorder	17 (8.5)	16 (7.8)	29 (7.1)
Bipolar disorder	37 (18.5)	36 (18.0)	73 (18.0)
Depression	54 (27.0)	63 (30.6)	117 (28.8)
Obsessive-compulsive disorder	10 (5.0)	12 (5.8)	22 (5.4)
Personality disorder	30 (15.0)	23 (11.2)	53 (13.1)
Schizoaffective disorder	10 (5.0)	19 (9.2)	29 (7.1)
Schizophrenia	36 (18.0)	41 (19.9)	78 (19.2)
Other diagnoses^2^	18 (9.0)	19 (9.2)	37 (9.)
Don’t know/Not answered	21 (10.5)	28 (13.6)	49 (12.7)
Marital status *n* (%)			
Single	103 (51.5)	103 (50.0)	206 (50.7)
Married	47 (23.5)	60 (29.1)	107 (26.4)
Separated/Divorced	45 (22.5)	41 (19.9)	86 (21.2)
Widowed	5 (2.5)	2 (1.0)	7 (1.7)
Educational level *n* (%)			
Primary not completed	9 (4.5)	3 (1.5)	12 (3.0)
Primary	61 (30.5)	67 (32.5)	128 (31.5)
Secondary	88 (44.0)	90 (43.7)	178 (43.8)
Higher	42 (21.0)	46 (22.3)	88 (21.7)
Living arrangement *n* (%)			
Original family	74 (37.0)	88 (42.7)	162 (39.9)
Own family	65 (32.5)	64 (31.1)	129 (31.8)
Alone	40 (20.0)	35 (11.0)	75 (18.5)
Shared flat	16 (8.0)	14 (6.8)	30 (7.4)
Other	5 (2.5)	5 (2.5)	10 (2.5)
Occupational status^1^ *n* (%)			
Caring for home and family	14 (7.0)	16 (7.8)	30 (7.4)
Disabled (receiving a pension)	133 (66.5)	140 (68.0)	273 (67.2)
Retired	7 (3.5)	8 (3.9)	16 (3,9)
Sick leave	13 (6.5)	5 (2.4)	18 (4.4)
Studying	4 (2.0)	8 (3.9)	12 (3.0)
Unemployed (with benefits)	27 (13.5)	31 (15.1)	58 (14.3)
Unemployed (without benefits)	5 (2.5)	5 (2.4)	10 (2.4)
Working	4 (2.0)	4 (1.9)	12 (3.0)
Other	–	2 (1.0)	2 (0.5)
Don’t know/Not answered	1 (0.5)	1 (0.5)	2 (0.5)

^1^ The categories are not mutually exclusive; people could choose more than one option

^2^ Attention deficit hyperactivity disorder, autism spectrum disorder, eating disorder, post-traumatic stress disorder, and f21–f29 diagnoses (ICD-10)

Participants did not receive any financial compensation. All procedures adhered to the ethical standards of the Declaration of Helsinki and its subsequent amendments The study was approved by the Bioethics Committee of the University of Barcelona (Institutional Review Board Number: IRB00003099).

### Statistical analysis

All data analyses were conducted using R 4.2.2, specifically with the packages lavaan [30] for CFA, lordif [31] for DIF analysis, irr for calculating ICC [32], and psych [33] for the remaining analyses.

In developing the short form, we used the development subsample (*n* = 200) to analyze data distribution at the item level, including median, skewness, and kurtosis. We also calculated the mean, standard deviation, and Shapiro-Wilk normality test for the total score.

To determine the adequacy of data for dimensionality analysis, we calculated the Kaiser-Meyer-Olkin (KMO) index and applied Bartlett’s test of sphericity. The KMO results were interpreted according to the criterion proposed by Howard [34] considering values above 0.70 as an acceptable range of variance. We then performed a one-factor exploratory factor analysis (EFA) using unweighted least squares (ULS) analysis for factor extraction. We used the ULS estimator for the EFA based on the polychoric correlation matrix, which has shown to provide more accurate and stable parameter estimates, and more precise standard errors than diagonally weighted least squares (DWLS), even under challenging conditions such as moderate to high skewness, low factor loadings, and a small number of indicators per factor [35]. Following the recommendations of Hair et al. [36] all items with a factor loading ≥ 0.70 were retained.

With the validation subsample (*n* = 206), we again analysed data distribution at the item level, including response frequency for each category, skewness, and kurtosis. We also calculated the mean, standard deviation, and the Shapiro-Wilk normality test for total scores on the short form of the Spanish NEL. A confirmatory factor analysis (CFA) was performed to confirm the unidimensional structure of the scale, using the weighted least squares mean and variance adjusted (WLSMV) estimator. Model fit was assessed using the chi-square test, the comparative fit index (CFI), the Tucker-Lewis index (TLI), and the standardized root mean squared residual (SRMR). According to Hu and Bentler [37], a CFI value ≥ 0.95, TLI ≥ 0.96, and SRMR ≤ 0.09 are indicative of adequate fit. We also report the chi-square/degrees of freedom ratio (*χ*^2^/df), with a ratio less than 3 being interpreted as indicating an acceptable fit of the data to the model [38].

Differential item functioning (DIF) by gender was explored using the ordinal logistic regression method [31]. For interpreting the effect size, which aids in understanding the magnitude and direction of DIF, we adhered to the recommendations of Jodoin and Gierl [39], whereby an effect size less than 0.035 indicates negligible DIF.

We also assessed the overall impact of DIF at the test level through differential test functioning (DTF) analysis, in this case using the approach proposed by Chalmers et al. [40], which involves calculating both signed DTF (*s*DTF) and unsigned DTF (*u*DTF). *s*DTF measures the direction and magnitude of DTF, indicating whether there is a systematic advantage or disadvantage for one group over another, while *u*DTF provides a measure of the total magnitude of DTF, regardless of direction. In both cases, we considered a value above 1 as a non-negligible difference.

Internal consistency for the total score on the short form of the Spanish NEL was assessed by calculating both McDonald’s omega (ω) and Cronbach’s alpha (α) for ordinal variables. Kline’s [41] recommendations were followed for interpreting internal consistency values, with coefficients over 0.90 considered excellent, 0.80 to 0.89 very good, and 0.70 to 0.79 adequate.

Temporal stability for the total score was estimated using the intraclass correlation coefficient (ICC), using the two-way mixed effects with absolute agreement, and interpreted according to the criteria of Koo and Li [42]: excellent if above 0.90, good from 0.75 to 0.89, moderate from 0.50 to 0.74, and poor if below 0.49.

To provide evidence of convergent validity, Spearman correlation coefficients were calculated to assess the relationship between scores on the short form of the Spanish NEL and other variables, by correlating its total score with the total scores on the MARS-12, DHS, and MSPSS. Given that empowerment, hope, and social support (connectedness) are key components of recovery according to CHIME framework [5] we expected positive correlations between the Spanish NEL scores and these measures: strong correlation with MARS-12 (i.e., recovery) and DHS (i.e., hope), and moderate correlation with MSPSS (i.e., social support) [43, 44]. Moreover, the total score on the Spanish NEL and its factors was compared with the total score on the short form of the same instrument.

Finally, to provide a preliminary guide for interpreting the Spanish NEL short-form scores for users of community mental health services, we calculated the percentile rank corresponding to each raw score using the total sample. We followed the standard procedure for transforming raw scores into percentile ranks [45], as well as the interpretation guidelines suggested by Cohen et al. [46]; that is, percentiles lower than 16 are indicative of below-average levels of empowerment, while percentiles greater than 84 indicate above-average levels.

## Results

### Phase 1: development of the Spanish NEL–Short form

Several items exhibited a significant negatively skewed distribution (items 1, 6, 12, 14, 16), with median values ranging between 4 and 5, indicating a tendency toward a higher level of empowerment. The Spanish NEL total score distribution deviated significantly from normality (*M* = 136.4, *SD* = 27.29; W = 0.99, *p* =.038).

Both the KMO index (0.85) and Bartlett’s test of sphericity (*χ*^2^(780) = 6498.465; *p* <.001) supported the adequacy of EFA, the purpose of which was to identify the items that best represented the empowerment construct. To this end, items with factor loadings ≥ 0.70 were retained for inclusion in the Spanish NEL–Short form. This selection process led to the inclusion of 12 items corresponding to five of the six factors of the original NEL, namely confidence and purpose, social support, caring community, connectedness, and self-management. The items selected for the Spanish NEL–Short form (hereinafter, Spanish NEL-12) are presented in Table 2. Detailed information on the item selection process is provided in Supplementary Table 1.

**Table 2:** Items selected for the Spanish NEL-12

No.	Spanish wording of item [English translation]	Factor loading
1	Tengo un propósito en mi vida [*I have a purpose in my life*].	0.77
2	Soy capaz de establecer mis límites [*I am able to set my boundaries*].	0.76
3	Sé qué hacer con los problemas que se me presentan [*I am able to deal with the problems that come my way*].	0.75
4	Estoy decidido/a a seguir adelante [*I am determined to go on*].	0.75
5	Tengo una vida estructurada [*I have structure in my life*].	0.73
6	Tengo una buena relación con las personas de mi entorno [*I have a good relationship with the people around me*].	0.73
7	La sociedad ofrece oportunidades para participar a mi manera [*This society creates opportunities that fit my level of participation*].	0.72
8	Siento que formo parte de algo [*I have a sense of belonging*].	0.72
9	Me valoro a mí mismo/a [*I think of myself as a person worth something*].	0.72
10	Hago cosas que son importantes para mí [*I do the things that I think are important*].	0.72
11	Puedo lidiar con mis vulnerabilidades [*I can deal with my vulnerabilities*].	0.71
12	Me atrevo a confiar en mí mismo/a [*I am not afraid to rely on myself*].	.70

### Phase 2: validation of the Spanish NEL-12

#### Item analysis

Item analysis of the Spanish NEL-12 indicated a negatively skewed distribution, with item 18 showing a value above − 1. The Shapiro-Wilk normality test showed that total scores on the Spanish NEL-12 were normally distributed (W = 0.99, *p* =.22; *M* = 40, *SD* = 9.44), while the Mardia test for multivariate normality suggested a non-normal distribution of the data (skewness = 1029.43, *p* <.001; kurtosis = 24.97, *p* <.001). Descriptive statistics for the Spanish NEL-12 are provided in Table 3.

**Table 3:** Descriptive statistics for the Spanish NEL-12

Item No.	M (SD)	S	K	W
1	3.32 (1.17)	−0.34	−0.71	0.91
2	3.17 (1.09)	−0.34	−0.37	0.90
3	3.04 (1.06)	−0.16	−0.31	0.90
4	4.04 (1.00)	−1.05	0.83	0.82
5	3.32 (1.13)	−0.44	−0.47	0.90
6	3.91 (0.89)	−0.75	0.60	0.85
7	3.10 (1.01)	−0.27	−0.31	0.90
8	3.40 (1.13)	−0.48	−0.50	0.90
9	3.11 (1.28)	−0.22	−1.07	0.90
10	3.68 (1.03)	−0.67	−0.03	0.87
11	2.99 (1.11)	−0.14	−0.64	0.91
12	3.21 (1.20)	−0.30	−0.77	0.91
Total score	40.29 (9.44)	−0.13	−0.38	0.99

*M*: mean, *SD*: standard deviation, *S*: skewness, *K*: kurtosis, *W*: Shapiro-Wilk normality test

#### Internal structure

**Fig. 1:**
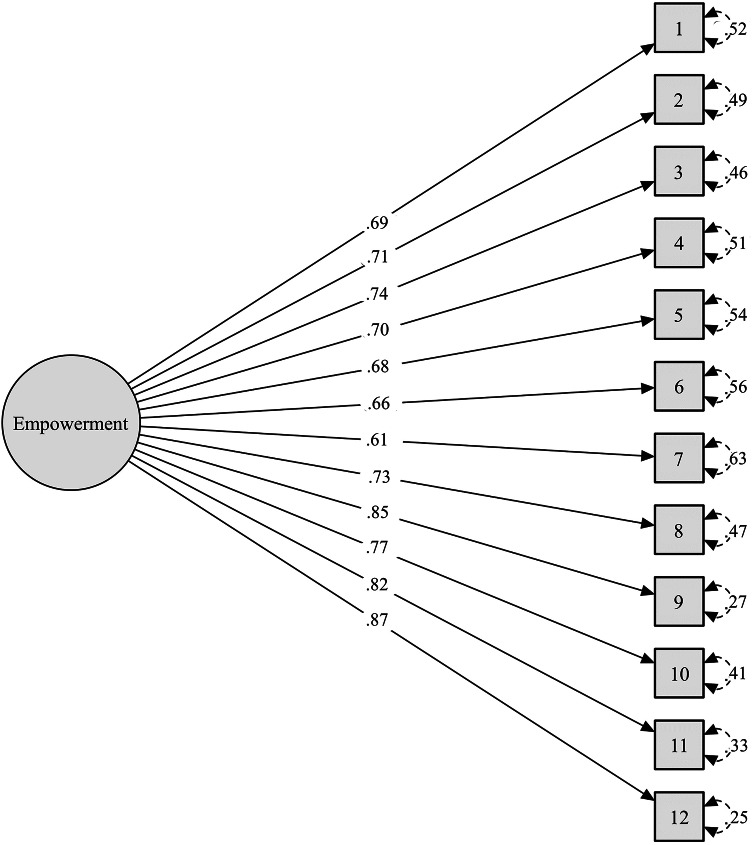
Path diagram of the confirmatory factor analysis of the Spanish NEL-12

CFA supported the one-factor model for the Spanish NEL-12 (*χ*^2^(54) = 158.22, *p* <.001, χ^2^/df = 2.93, CFI = 0.97, TLI = 0.97, SRMR = 0.050). Factor loadings ranged from 0.61 to 0.87 and were significant for all items (*p* <.001). The path diagram is shown in Fig. 1.

**Fig. 2:**
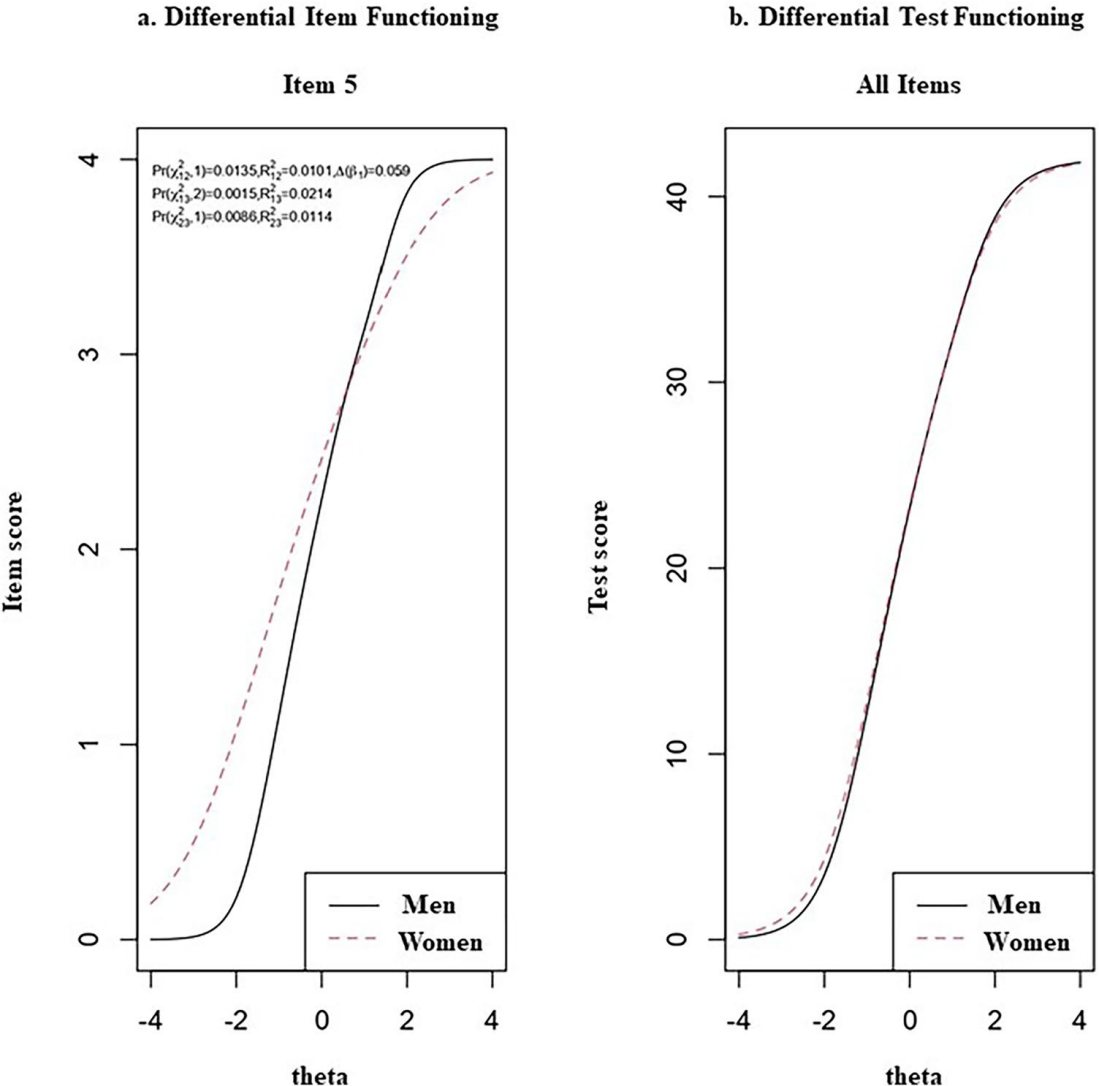
Detection of differential item functioning by gender

Using ordinal logistic regression, we identified gender-based DIF in item 5 of the Spanish NEL-12 (equivalent to item 20 of the Spanish NEL). As Fig. 2a shows, the effect size measured with McFadden’s *R*^2^ was less than 0.035, indicating negligible DIF. We confirm this negligible DIF in Fig. 2b, where test characteristic curves (TCCs) for women and men were traced for all items at each level of empowerment. These curves show that there is minimal difference between women and men in the overall test score.

We also analyzed DTF to examine whether the DIF displayed by item 5 affected the total score on the Spanish NEL-12. The values of both *s*DTF (0.118; 95% CI: −0.71 to 0.82) and *u*DTF (0.581; 95% CI: 0.31 to 1.89) indicated that no DTF was detectable across the entire range of empowerment measurement with the Spanish NEL-12. The former value (0.118) means that test scoring is biased by only a small average amount, and the absolute deviations over the whole test indicated a small (0.581) average area between curves. These results confirm what is shown in Fig. 2b.

#### Internal consistency and Temporal stability

The Spanish NEL-12 total score demonstrated excellent internal consistency (ω = 0.94, 95% CI [0.92, 0.96]; α = 0.93, 95% CI [0.86, 0.98]) and temporal stability (ICC = 0.86 [0.77, 0.91]).

#### Validity evidence based on relationships with other variables

Scores on the Spanish NEL-12 showed a strong positive correlation with scores on both the MARS-12 (*n* = 176, *r*_*s*_ = 0.82, *p* <.001) and the DHS (*n* = 176, *r*_*s*_ = 0.81, *p* <.001), and a moderate positive correlation with scores on the MSPSS (*n* = 175, *r*_*s*_ = 0.59, *p* <.001). Scores on the Spanish NEL-12 were also strongly and positively correlated with the Spanish NEL total score (*n* = 206, *r*_*s*_ = 0.95, *p* <.001), as well as with scores on the factors confidence and purpose (*r*_*s*_ = 0.94, *p* <.001), social support (*r*_*s*_ = 0.69, *p* <.001), connectedness (*r*_*s*_ = 0.85, *p* <.001), and self-management (*r*_*s*_ = 0.86, *p* <.001). A moderate positive correlation was found with scores on the caring community factor (*r*_*s*_ = 0.57, *p* <.001), while the correlation with the professional help factor was lower (*r*_*s*_ = 0.46, *p* <.001).

### Percentile rank scores

Table 4 shows the percentile ranks corresponding to each raw score on the Spanish NEL-12, based on our sample. Raw scores lower than 28 indicate a below-average level of empowerment, whereas scores higher than 49 suggest an above-average level of empowerment.

**Table 4:** Percentile scores of the Spanish NEL-12 in community mental health services users

Score	Percentile	Score	Percentile
12	1	37	42
13	1	38	45
14	1	39	51
15	1	40	54
16	1	41	56
17	2	42	59
18	2	43	62
19	3	44	66
20	4	45	70
21	5	46	73
22	5	47	77
23	6	48	81
24	7	49	83
25	9	50	86
26	11	51	89
27	13	52	91
28	16	53	93
29	18	54	95
30	20	55	96
31	22	56	97
32	24	57	98
33	27	58	98
34	31	59	99
35	34	60	99
36	38		

## Discussion

To our knowledge, this represents the first attempt to develop a short form of the NEL. In terms of content coverage, a comparison of the items retained in the Spanish NEL-12 with the original six-factor Dutch scale [11] shows that five of these factors (namely, confidence and purpose, social support, self-management, connectedness, and caring community) are represented in the items of our one-factor short form. The only exception was the professional help factor, which had no items with a factor loading over 0.70. It should be noted here that in the original validation study of the Spanish NEL, two of the four items from this factor yielded some of the lowest factor loadings, and its scores were not significantly correlated with any of the other scales implemented [19]. Furthermore, previous studies that have evaluated this dimension not only found that professional help does not predict greater empowerment but also suggested an inverse correlation: the more individuals use traditional mental health services, the less empowered they tend to be [14].

The validation phase of the present study confirmed the unidimensionality of the Spanish NEL-12, and the high and statistically significant standardized factor loadings underscore its effectiveness in measuring a single underlying attribute: empowerment. Additionally, DIF and DTF analyses revealed that the items of the Spanish NEL-12 are invariant across genders, indicating that the scale measures empowerment similarly for both men and women. Accordingly, researchers may use the total score of the Spanish NEL-12 to examine differences and similarities in empowerment levels without fear of gender-related measurement bias; in other words, any differences in scores between groups of men and women would reflect true differences in empowerment levels rather than bias [47]. The ability of the Spanish NEL-12 to effectively measure empowerment levels across genders is essential for identifying and addressing gender disparities in mental health outcomes. As such, the availability of this tool can make an important contribution to the development and implementation of targeted policies and practices aimed at reducing these disparities.

Reliability analysis demonstrated high temporal stability of scores on the Spanish NEL-12 after one to two weeks, along with excellent internal consistency. Furthermore, these values closely resembled those obtained with the 40-item Spanish NEL, with both Spanish scales yielding slightly higher coefficients than was the case in the Dutch sample.

In terms of validity evidence based on relationships with other variables, scores on the Spanish NEL-12 were also strongly correlated with scores on other scales that assess components of the CHIME framework [5], specifically hope (the DHS) [24] and perceived social support (the MSPSS) [25, 26], as well as with scores on a measure of personal recovery (the MARS-12) [23]. This highlights the integral role of empowerment in the recovery process, due to its importance at both the individual and societal levels. At the individual level, empowerment is what enables a person to gain greater control over their life and to participate more actively in the recovery process, with this being achieved through enhanced self-esteem and greater self-determination and autonomy. At the societal level, empowerment is crucial for establishing social networks, mobilizing social support, and exerting greater influence on social and political decision-making processes [1].

Furthermore, the Spanish NEL-12 exhibited good psychometric properties in terms of internal structure, internal consistency, temporal stability, and relationships with other variables, with the results being very similar to those obtained with the 40-item scale. Thus, despite the exclusion of items referring to professional help, the Spanish NEL-12 effectively balances content coverage and test length, while maintaining robust psychometric properties. It therefore meets the high standards expected of a short form, providing an efficient alternative to the original test [48] as a measure of empowerment among Spanish-speaking users of mental health services.

The present study has certain limitations that must be acknowledged. The most notable is the use of convenience sampling, with all participants being users of community mental health services. This kind of service offers professional support to promote social functioning and community inclusion among individuals who, while diagnosed with a mental disorder, are considered clinically compensated. Future studies should therefore seek to validate the Spanish NEL-12 in more diverse populations and settings so as to further confirm its utility and robustness across different contexts. Another potential limitation is that items related to the professional help factor were excluded from the Spanish NEL-12 due to their low factor loadings. As a result, the scale may not fully capture aspects of empowerment linked to relationships with professionals, which could be an important component of some users’ empowerment experiences.

Despite these limitations, the study makes an important contribution by developing the first short form of the NEL and providing preliminary percentile rank scores for the Spanish NEL-12 in community mental health services users. Although these scores are specific to individuals with characteristics similar to those of the current sample, they offer clinicians and researchers in Spanish-speaking countries an initial reference point for assessing empowerment among mental health service users.

## Conclusions

This study has successfully developed and validated a short form of an instrument for measuring empowerment in mental health. Our findings suggest that the Spanish NEL-12 provides valid and reliable scores, and they support its use as an empowerment assessment tool with Spanish-speaking users of community mental health services. The Spanish NEL-12 offer a significant advantage in clinical and research settings where the longer version may be impractical due to time constraints or respondent fatigue. Consequently, this streamlined tool not only broadens the accessibility of empowerment measurement but also enhances the feasibility of incorporating its assessment into routine mental health care and research.

## Supplementary Information

Below is the link to the electronic supplementary material.

Supplementary Material 1

## Data Availability

The data that support the findings of this study are available from the corresponding author upon reasonable request.
