# Supplementary material for: A short form of the Spanish version of the Netherlands empowerment list: development and validation
Source: Soc Psychiatry Psychiatr Epidemiol. 2025 Jul 30;61(3):575–85. doi: 10.1007/s00127-025-02970-9 (PMC12996410; doi:10.1007/s00127-025-02970-9)
Supplement: Supplementary file 1 — Supplementary Material 1 [file 127_2025_2970_MOESM1_ESM.docx]

**Supplementary Table 1**

Descriptive statistics and factor loadings for items in the Spanish NEL and the Spanish NEL-12.

| Spanish NEL Item No. | | Spanish NEL-12 Item No | *M (SD)* | *S* | *K* | Factor  loading | | Communality |
| --- | --- | --- | --- | --- | --- | --- | --- | --- |
| 1 |  |  | 4.35 (0.86) | -1.52 | 2.54 | .43 |  | .18 |
| 2 |  |  | 2.49 (1.14) | 0.51 | -0.49 | .48 |  | .23 |
| 3 |  |  | 3.55 (1.11) | -0.59 | -0.29 | .47 |  | .22 |
| 4 |  | 1 | 3.16 (1.34) | -0.17 | -1.16 | .72 |  | .52 |
| 5 |  |  | 3.84 (1.06) | -0.69 | -0.05 | .61 |  | .37 |
| 6 |  |  | 4.07 (1.02) | -1.04 | 0.66 | .34 |  | .12 |
| 7 |  | 2 | 3.15 (1.11) | -0.15 | -0.77 | .73 |  | .53 |
| 8 |  |  | 3.69 (1.03) | -0.62 | 0.04 | .65 |  | .42 |
| 9 |  |  | 3.66 (1.03) | -0.35 | -0.47 | .66 |  | .43 |
| 10 |  |  | 3.57 (1.14) | -0.53 | -0.66 | .64 |  | .41 |
| 11 |  |  | 2.76 (1.28) | 0.25 | -0.97 | .56 |  | .31 |
| 12 |  |  | 4.23 (1.10) | -1.62 | 1.98 | .54 |  | .29 |
| 13 |  | 3 | 2.74 (1.08) | 0.05 | -0.79 | .75 |  | .56 |
| 14 |  |  | 4.28 (0.91) | -1.46 | 2.18 | .54 |  | .29 |
| 15 |  |  | 3.21 (1.17) | -0.09 | -0.75 | .60 |  | .36 |
| 16 |  |  | 4.07 (1.05) | -1.17 | 0.98 | .65 |  | .42 |
| 17 |  |  | 3.78 (1.04) | -0.74 | 0.18 | .67 |  | .45 |
| 18 |  | 4 | 3.93 (1.15) | -0.82 | -0.30 | .71 |  | .50 |
| 19 |  |  | 3.14 (1.17) | -0.18 | -0.78 | .61 |  | .37 |
| 20 |  | 5 | 3.09 (1.19) | -0.27 | -0.84 | .72 |  | .51 |
| 21 |  |  | 3.11 (1.21) | -0.10 | -0.94 | .60 |  | .36 |
| 22 |  |  | 3.76 (1.13) | -0.83 | -0.12 | .60 |  | .35 |
| 23 |  |  | 2.99 (1.22) | -0.03 | -0.87 | .63 |  | .40 |
| 24 |  |  | 2.78 (1.35) | 0.17 | -1.21 | .58 |  | .34 |
| 25 |  |  | 3.69 (1.17) | -0.71 | -0.41 | .50 |  | .25 |
| 26 |  |  | 3.56 (1.16) | -0.51 | -0.53 | .56 |  | .31 |
| 27 |  | 6 | 3.87 (0.99) | -0.70 | 0.01 | .72 |  | .52 |
| 28 |  | 7 | 3.05 (1.13) | -0.23 | -0.62 | .70 |  | .49 |
| 29 |  |  | 3.77 (1.14) | -0.87 | 0.04 | .62 |  | .39 |
| 30 |  | 8 | 3.37 (1.30) | -0.40 | -0.97 | .72 |  | .52 |
| 31 |  | 9 | 2.96 (1.33) | -0.05 | -1.19 | .77 |  | .59 |
| 32 |  |  | 2.62 (1.24) | 0.27 | -0.96 | .66 |  | .44 |
| 33 |  |  | 3.63 (1.13) | -0.72 | -0.10 | .30 |  | .09 |
| 34 |  |  | 3.74 (1.26) | -0.83 | -0.36 | .65 |  | .42 |
| 35 |  |  | 3.49 (1.22) | -0.41 | -0.87 | .69 |  | .47 |
| 36 |  |  | 2.36 (1.17) | 0.55 | -0.62 | .50 |  | .25 |
| 37 |  | 10 | 3.59 (1.10) | -0.42 | -0.58 | .73 |  | .53 |
| 38 |  | 11 | 2.74 (1.13) | 0.10 | -0.84 | .76 |  | .58 |
| 39 |  |  | 3.69 (1.11) | -0.63 | -0.33 | .61 |  | .37 |
| 40 |  | 12 | 2.95 (1.29) | -0.07 | -1.09 | .75 |  | .56 |

*M:* mean, *SD:* standard deviation, *S*: skewness, *K*: kurtosis.
